# Anti–SARS-CoV Immunoglobulin G in Healthcare Workers, Guangzhou, China

**DOI:** 10.3201/eid1101.040138

**Published:** 2005-01

**Authors:** Wei-Qing Chen, Ci-Yong Lu, Tze-Wai Wong, Wen-Hua Ling, Zhong-Ning Lin, Yuan-Tao Hao, Qing Liu, Ji-Qian Fang, Yun He, Fu-Tian Luo, Jin Jing, Li Ling, Xiang Ma, Yi-Min Liu, Gui-Hua Chen, Jian Huang, Yuan-Sen Jiang, Wen-Qi Jiang, He-Qun Zou, Guang-Mei Yan

**Affiliations:** * Sun Yat-Sen University, Guangzhou, People’s Republic of China; † Chinese University of Hong Kong, Hong Kong Special Administrative Region, People’s Republic of China

**Keywords:** SARS, Seroprevalence, Healthcare workers, China, research

## Abstract

To determine the prevalence of inapparent infection with severe acute respiratory syndrome (SARS) among healthcare workers, we performed a serosurvey to test for immunoglobulin (Ig) G antibodies to the SARS coronavirus (SARS-CoV) among 1,147 healthcare workers in 3 hospitals that admitted SARS patients in mid-May 2003. Among them were 90 healthcare workers with SARS. As a reference group, 709 healthcare workers who worked in 2 hospitals that never admitted any SARS patients were similarly tested. The seroprevalence rate was 88.9% (80/90) for healthcare workers with SARS and 1.4% (15/1,057) for healthcare workers who were apparently healthy. The seroprevalence in the reference group was 0.4% (3/709). These findings suggest that inapparent infection is uncommon. Low level of immunity among unaffected healthcare workers reinforces the need for adequate personal protection and other infection control measures in hospitals to prevent future epidemics.

## Abstract 2

Low level of immunity for SARS-CoV among well healthcare workers reinforces the need for infection control measures in hospitals to prevent epidemics.

On January 2, 2003, a patient was admitted to the Traditional Medicine Hospital, Guangdong Province, with fever, cough, decreased leukocyte count, and abnormal chest radiographs. Shortly after the patient was admitted, 7 healthcare workers who cared for him became ill with similar symptoms. This index patient was retrospectively confirmed to be the first patient with severe acute respiratory syndrome (SARS) in Guangzhou (*1*). Subsequently, outbreaks of SARS occurred in several hospitals (*2*), and the disease rapidly spread to the Guangzhou community (*1*). In mid-February, the incidence of SARS reached a peak and gradually declined thereafter. When the last case was reported on May 9, 2003, a total of 1,284 probable SARS cases had been reported in Guangzhou (*3*).

In most cases, the disease was spread through close contact with an infected person (*4*). **A** high incidence of SARS was observed among healthcare workers, especially during the early stages of the SARS epidemic (*5*–*8*); healthcare workers were likely exposured to the SARS coronavirus (SARS-CoV) at the workplace. Also, SARS-CoV can survive for many hours on environmental surfaces **(***5***); therefore**, infection through contact with contaminated fomites is a distinct possibility, despite the absence of supportive epidemiologic evidence. A series of strict preventive measures, including specially designed wards to accommodate SARS patients and the use of gloves, eye protection, face masks, foot covers, and protective gowns, were adopted to control the spread of SARS to healthcare workers. Whether inapparent infections existed with this new epidemic was unclear. In this study, we explored the seroprevalence of antibodies to SARS-CoV in healthcare workers who had cared for SARS patients but did not have SARS and those working in hospitals with no SARS patients. We also determined the seroprevalence of antibodies to SARS-CoV in convalescent healthcare workers in whom SARS was diagnosed.

## Methods

### Study Populations

In mid-May 2003, ≈4 months after the initial SARS outbreak in Guangzhou, a cross-sectional survey was carried out on healthcare workers who worked with SARS patients in the First, Second ,and Third Affiliated Hospitals of the Sun Yat-Sen University, Guangzhou. Outbreaks of SARS had occurred among healthcare workers in the Second and Third Affiliated Hospitals but not the First Affiliated Hospital. Of the 1,394 healthcare workers who cared for SARS patients in these 3 hospitals, 1,147 (82.3%) were surveyed. Those surveyed included healthcare workers from all departments that cared for SARS patients. All healthcare workers on duty were surveyed; only those who were off-duty during the survey were excluded.

For comparison, 709 healthcare workers were sampled from 2 hospitals with no SARS patients: the Affiliated Cancer Hospital, Guangzhou, where no SARS patients were admitted, and the Fifth Affiliated Hospital, Zhuhai, where no SARS cases were reported in the community. A total of 1,856 healthcare workers were surveyed.

### Serum Collection and Interview

For each healthcare worker, 10 mL of peripheral venous blood was collected; the serum was separated and stored at –70°C. A standardized interview with a structured questionnaire was used to obtain information on sociodemographic characteristics and the history of SARS according to a case definition of SARS by the Ministry of Health, China (*9*).

A probable case-patient was defined as one who had close contact with a patient, was a member of an infected cluster, or infected other persons; had symptoms and signs of febrile respiratory symptoms, and had changes on chest radiograph. A patient was also considered to have a probable case if he or she visited or lived in an area where SARS was reported with secondary transmission within 2 weeks of illness onset, had symptoms and signs of febrile respiratory illness, had normal or decreased leukocyte count, and had changes on chest radiograph.

A suspected case-patient was defined as one who had close contact with a patient; was a member of an infected cluster, or infected other persons; and had symptoms and signs of febrile respiratory illness; and had normal or decreased leukocyte count. A patient was also considered to have a suspected case if he or she visited or lived in an area where SARS was reported with secondary transmission within 2 weeks of illness onset, had symptoms and signs of febrile respiratory illness, and had changes on chest radiograph. If a patient had no epidemiologic link to SARS but he or she had symptoms and signs of febrile respiratory illness, normal or decreased leukocyte count, and changes on chest radiograph, he or she was still considered to have a suspected case.

A person who had visited or lived in cities where SARS cases were reported with secondary transmission was placed under medical observation if he or she had symptoms and signs of febrile respiratory illness and had a normal or decreased leukocyte count.

### Detecting Serum IgG against SARS-CoV

Immunoglobulin (Ig) G against SARS-CoV were detected by enzyme-linked immunosorbent assay (ELISA) according to the manufacturer’s instructions (Beijing BGI-GBI Biotech Co., Ltd.) (*10*). This ELISA has a sensitivity of 89.9% and a specificity of 99.0% (*11*).

### Statistical Analysis

Means and standard deviations were used to describe continuous variables. Proportions and prevalence rates were used to describe categorical variables. Chi-square tests were performed to test the association between SARS-CoV IgG seropositivity and the sociodemographic characteristics of the healthcare workers.

## Results

### Sociodemographic Characteristics

The mean age of the healthcare workers investigated was 30.78 years (SD 9.1 years); 71.6% were women. Details of their sociodemographic characteristics are presented in Table 1. General information related to SARS in the 5 hospitals is shown in Table 2.

**Table 1:** Sociodemographic characteristics of 1,856 healthcare workers

Sociodemographic characteristic	No. participants (%)
Hospital*	
First Affiliated Hospital	389 (21.0)
Second Affiliated Hospital	361 (19.5)
Third Affiliated Hospital	397 (21.4)
Affiliated Cancer Hospital	371 (20.0)
Fifth Affiliated Hospital	338 (18.1)
Age (y)	
<26	676 (36.5)
26–30	404 (21.8)
31–35	294 (15.8)
36–40	221 (11.9)
>40	261 (13.0)
Sex	
Male	528 (28.4)
Female	1,328 (71.6)
Educational level	
Senior school	136 (7.3)
Technical secondary school	718 (38.7)
Junior college	367 (19.8)
University	333 (17.9)
MD/PhD	302 (16.3)
Department	
SARS ward	413 (22.3)
Emergency department/fever clinic	196 (10.6)
Infectious disease department	125 (6.7)
Respiratory diseases department	101 (5.4)
ICU	61 (3.3)
X-ray	74 (4.0)
Laboratory	66 (3.6)
Others†	820 (44.2)
Job title‡	
Doctor	567 (30.7)
Nurse	892 (48.3)
Health attendant	101 (5.5)
Technician in laboratory	74 (4.0)
Others	213 (11.5)

*All 5 hospitals are teaching hospitals of the Sun Yat-Sen University. †Departments of internal medicine, surgery, and logistic service. ‡Missing for 9 healthcare workers.

**Table 2:** Severe acute respiratory syndrome (SARS)-related information in the 5 affiliated hospitals

SARS-related information	First Affiliated Hospital	Second Affiliated Hospital	Third Affiliated Hospital	Affiliated Cancer Hospital	Fifth Affiliated Hospital
In SARS-epidemic area?	Yes	Yes	Yes	Yes	No
No. healthcare workers exposed to SARS*	548	421	425	0	0
No. healthcare workers surveyed	389	361	397	371	338
No. days when SARS patients were in the hospital	120	110	102	0	0
No. probable SARS patients cared for	122	150	31	0	0
No. suspected SARS patients cared for	102	50	30	3	0
No. SARS patients who required tracheal intubation	10	0	1	0	0
No. SARS patients who required tracheotomy	0	1	2	0	0
Cared for the index patient?†	No	Yes	Yes	0	0
No. healthcare workers who had SARS‡ in SARS wards	0	80	20	0	0
No. healthcare workers who had from SARS in non-SARS wards	3	10	2	0	0

*This refers to healthcare workers caring for SARS patients and laboratory personnel handling specimens from SARS patients. †The index patient was identified as a superspreader who subsequently infected >100 persons (both healthcare workers and other patients and family members in the Second Hospital and Third Hospital). ‡According to the clinical and epidemiologic case definition.

Prevalence of IgG against SARS-CoV among Healthcare Workers

Among healthcare workers working with SARS patients, the prevalence of IgG against SARS-CoV was 88.9% (80/90) for those who contracted SARS and 1.4% (15/1,057) for those who did not (Table 3). By contrast, the seroprevalence was 0.5% (2/371) for healthcare workers working in the non-SARS hospital in Guangzhou and 0.3% (1/338) for healthcare workers in the hospital in SARS-free Zhuhai. The overall seroprevalence in this reference group of healthcare workers was 0.4% (3/709).

**Table 3:** Severe acute respiratory (SARS) immunoglobulin G prevalence among healthcare workers with and without SARS in 5 affiliated hospitals

Affiliated hospital	Healthcare workers with SARS		Healthcare workers without SARS	
	No. participants	No. positive (%)	No. participants	No. positive (%)
First	0	0	389	4 (1)
Second	73	63 (86.3)	288	8 (2.8)
Third	17	17 (100)	380	3 (0.8)
Cancer	0	0	371	2 (0.5)
Fifth	0	0	338	1 (0.3)
Total/overall percentage	90	80 (88.9)	1,766	18 (1)

We also compared the prevalence of anti-SARS IgG in healthcare workers for each sociodemographic characteristic. We analyzed the data on healthcare workers who worked with SARS patients in the 3 hospitals. These results are presented in Table 4.

**Table 4:** Severe acute respiratory syndrome (SARS) immunoglobulin G prevalence for different sociodemographic characteristics

Sociodemographic characteristics	No. participants	No. positive for IgG	Prevalence (%)
Age (y)*			
<26	355	44	12.4
26–30	310	17	5.5
31–35	211	14	6.6
36–40	118	9	7.6
>40	141	11	7.8
Sex†			
Male	306	15	4.7
Female	743	80	9.7
Educational level*			
Senior school	112	14	12.5
Technical secondary school	401	42	10.5
Junior college	210	11	5.2
University	197	17	8.6
MD/PhD	227	11	4.8
Department‡			
SARS ward	409	13	3.2
Emergency/fever diagnoses	188	4	2.1
Infection	125	19	15.2
Respiratory	100	36	36.0
ICU	55	7	12.7
X-ray	57	2	3.5
Laboratory	66	0	0.0
Others	147	14	9.5
Job title†			
Doctor	388	24	6.2
Nurse	510	52	10.2
Healthcare attendants	91	12	13.2
Technician in laboratory	66	0	0.0
Others§	92	7	7.6

*p < 0.05. †p < 0.01. ‡p < 0.001. §Department of internal medicine, surgery and logistic service.

The results showed that the seroprevalence of anti-SARS IgG in healthcare workers <26 years of age was significantly higher than in those >26 years of age (p < 0.05). Women had a higher seroprevalence than men (p < 0.01). Those with a senior school or technical secondary school education had a higher seroprevalence than those with tertiary education. Seroprevalence was highest among healthcare workers working in departments of respiratory diseases, followed by those in departments of infectious diseases, then in intensive care units; the prevalence was <10% in all remaining departments (p < 0.001). No laboratory personnel had IgG against SARS. When healthcare workers were compared to those with senior positions, those at a more junior level had a higher risk for infection by SARS-CoV (p < 0.01).

## Discussion

Anti-SARS IgG can be detected 1–2 weeks after the onset of symptoms. Almost all SARS patients in the convalescent stage had anti-SARS IgG in their serum samples (*11*–*14*). In our study, all healthcare workers with SARS were in the convalescent stage, and SARS-CoV infected most while they were caring for the same index patient who was subsequently identified as a superspreader (*15*). The finding of a 100% seroprevalence of SARS IgG among 17 SARS-infected healthcare workers in the Third Affiliated Hospital was identical to the results by Li et al. (*12*), who tested SARS IgG at different stages among the same group of SARS-infected healthcare workers by using the same ELISA. By contrast, 63 (86.3%) of 73 healthcare workers with SARS in the Second Affiliated Hospital were seropositive for SARS IgG. Some of these healthcare workers might have been misdiagnosed, as the clinical diagnosis of SARS was not specific (*16*). Even allowing for this possibility, the overall high seropositivity rate of 88.9% among SARS patients is similar to findings by Wang et al. in Beijing, who used the same test (*11*); Chow et al. in Singapore, who used a different EIA (*17*); and Chan et al. in Hong Kong, who used an immunofluorescence assay (*18*). All of these studies indicate that serum IgG antibodies to SARS-CoV at the convalescent stage of the illness can be useful in confirming the disease.

The low seroprevalence o SARS IgG (0.3%–2.8%) in healthy healthcare workers with different levels of exposure to SARS patients is similar to that reported by Wang et al. (*11*). However, a similar study by Chow et al. in Singapore did not find any serologic evidence of subclinical infection among a population with a high likelihood of exposure to the virus. Our ELISA was 99% specific (*11*). This specificity could have produced a few false-positive results, which accounts for a low seropositive rate of 0.4% (3/709) among healthcare workers in the reference group, who had no exposure to SARS in their hospitals. One healthcare worker in Zhuhai, where no SARS occurred, was seropositive, which could be a false-positive result. However, we could not exclude the possibility of inapparent infection among healthcare workers in the 4 hospitals in Guangzhou. Another possibility is cross-reaction with other human coronaviruses. A more specific test, such as the indirect immunofluorescence test, should clarify this uncertainty (*18*).

The low seroprevalence of SARS IgG, at 1.4% (15/1,057) among apparently healthy frontline healthcare workers in all 3 SARS hospitals, suggests that inapparent infection is relatively uncommon. We did not, however, ascertain whether the healthcare workers with a positive antibody test result were carriers of SARS-CoV. Overall, the low seropositivity among healthy healthcare workers suggests that the level of immunity to SARS in the general population in Guangzhou was too low to constitute an effective immune barrier against the spread of SARS. Should the disease recur there, every effort should be made to protect healthcare workers and the general public from being infected by SARS patients.

The First Affiliated Hospital only admitted SARS patients after outbreaks had occurred among healthcare workers in the Second and Third Affiliated Hospitals. After these outbreaks, a series of protective measures were adopted in all 3 hospitals. Sufficient preparation, such as personal protection and designated SARS wards, is important to avert hospital outbreaks. The low seroprevalence of SARS IgG among healthcare workers working in the First Affiliated Hospital indicated the effectiveness of these measures. This finding is consistent with the study by Chow et al. (*17*).

The seroprevalence rates were significantly different among the healthcare workers who cared for SARS patients when classified by their age, sex, educational level, hospital, department, and job title. These differences could be due to the probability of exposure to the SARS index case. On January 30, 2003, the index patient was admitted to the Department of Respiratory Diseases of the Second Affiliated Hospital. On February 1, he was transferred to the Third Affiliated Hospital because of worsening dyspnea. During his stay in these 2 hospitals, where protective measures were lacking, he directly and indirectly infected 90 healthcare workers and 22 healthcare workers in the Second and Third Affiliated Hospitals, respectively. This finding accounts for the much higher seroprevalence of SARS IgG among healthcare workers in the departments of respiratory diseases and infectious diseases. Healthcare workers in SARS wards and fever clinics were fully equipped with personal protective measures (caps, gowns, multilayered cotton face masks, eye shields, gloves, and foot covers), which might explain their much lower seroprevalence. None of our laboratory healthcare workers, who performed serologic tests but not live viral tests, were seropositive, which suggests that the probability of SARS infection by handling serum samples of SARS patients was low. Of all occupations, healthcare attendants had the highest seropositive rate, which might be related to their general lower level of education and a lack of training in infection control measures. Future efforts to improve SARS containment should also address this problem among nonprofessional staff.

This study has several limitations. Even though the response rate among healthcare workers who cared for SARS patients was high (82.3%), some selection bias is inevitable. Moreover, the study was limited to 5 university hospitals, so we caution against the extrapolation of our findings to healthcare workers in other hospitals and to the general population. We have not tested serum samples from our healthcare workers against other pathogens, e.g., *Mycoplasma pneumoniae* and influenza virus, which limits our ability to exclude the nonspecific and atypical pneumonia caused by these agents (*16*).

In conclusion, this study shows that a high proportion of healthcare workers who have contracted SARS have IgG against SARS-CoV in their serum samples after they have fully recovered. Inapparent infection with SARS is uncommon. The low seropositivity against SARS among healthcare workers who have not been exposed to SARS patients suggests a lack of immunity in this group and in the general population, where the number of SARS cases is comparatively small.
